# Li-Assisted Low-Temperature Phase Transitions in Solution-Processed Indium Oxide Films for High-Performance Thin Film Transistor

**DOI:** 10.1038/srep25079

**Published:** 2016-04-28

**Authors:** Manh-Cuong Nguyen, Mi Jang, Dong-Hwi Lee, Hyun-Jun Bang, Minjung Lee, Jae Kyeong Jeong, Hoichang Yang, Rino Choi

**Affiliations:** 1 Department of Materials Science and Engineering, Inha University, Incheon 402-751, Republic of Korea; 2 Department of Applied Organic Materials Engineering, Inha University, Incheon 402-751, Republic of Korea; 3 Department of Electronic Engineering, Hanyang University, Seoul 133-791, Republic of Korea

## Abstract

Lithium (*Li*)-assisted indium oxide (*In*_*2*_*O*_*3*_) thin films with ordered structures were prepared on solution-processed zirconium oxide (*ZrO*_*2*_) gate dielectrics by spin-casting and thermally annealing hydrated indium nitrate solutions with different *Li* nitrate loadings. It was found that the *Li*-assisted *In* precursor films on *ZrO*_*2*_ dielectrics could form crystalline structures even at processing temperatures (*T*) below 200 °C. Different *In* oxidation states were observed in the *Li*-doped films, and the development of such states was significantly affected by both temperature and the mol% of *Li* cations, [*Li*^+^]/([*In*^*3*+^] + [*Li*^+^]), in the precursor solutions. Upon annealing the *Li*-assisted precursor films below 200 °C, metastable indium hydroxide and/or indium oxyhydroxide phases were formed. These phases were subsequently transformed into crystalline *In*_*2*_*O*_*3*_ nanostructures after thermal dehydration and oxidation. Finally, an *In*_*2*_*O*_*3*_ film doped with 13.5 mol% *Li*^+^ and annealed at 250 °C for 1 h exhibited the highest electron mobility of 60 cm^2^ V^−1^ s^−1^ and an on/off current ratio above 10^8^ when utilized in a thin film transistor.

Metal oxide semiconductors (*MOSs*) such as indium gallium zinc oxide (*IGZO*), indium zinc oxide (*IZO*), zinc tin oxide (*ZTO*), and indium oxide (*In*_*2*_*O*_*3*_) have been extensively studied for use as active channel materials in thin film transistors (TFTs) for flat panel displays^1^,^2^,^3^,^4^,^5^,^6^,^7^,^8^,^9^,^10^,^11^,^12^,^13^,^14^. Such interest can be attributed to the reasonably high carrier mobility, stability, and large-area uniformity of these compounds. Furthermore, when compared to polycrystalline silicon, the lower processing cost associated with *MOS* thin films has made these materials more attractive for large-area display applications.

Among the various methods for preparing *MOS* films, solution-based approaches are particularly appealing due to their simplicity and the ease with which stoichiometry can be controlled^4^,^5^,^6^,^7^,^8^. However, the annealing temperature (*T*_A_) utilized in the synthesis of *MOS* layers tends to be determined by the softening *T* of the supporting substrate. The use of an undesirably low *T*_A_ in the processing of *MOS* precursors leads to the presence of residual carbon impurities and a lower degree of domain ordering in the resulting films. Such structures subsequently exhibit degraded electrical properties when assisted into TFTs^3^. In an effort to enhance the semiconducting properties of solution-processed *MOS* nanostructures while maintaining an *T*_A_ below 400 °C, several processing strategies have been devised, including combustion synthesis, the use of additional doping and seeding layers^5^,^15^,^16^,^17^, and the introduction of preformed nanocrystals into precursor solutions^4^,^9^.

Recently, solution-processed *In*_*2*_*O*_*3*_- and *ZnO*-based TFTs have been intensively studied due to their high electron mobility (*μ*_e_) and low background conductivity^2^,^3^,^4^,^5^,^6^,^7^,^8^,^9^,^10^,^11^,^12^,^13^,^14^,^15^,^16^,^17^. Particular attention has been focused on the development of a novel low-*T* solution-based preparation procedure that does not degrade the electrical performance of *In*_*2*_*O*_*3*_ TFTs. Significant progress has already been achieved in this area, as *In*_*2*_*O*_*3*_ TFTs with *μ*_e_ values of ~4.0 cm^2^ V^−1^ s^−1^ (*T*_A_ = 200 °C) on SiO_2_ dielectrics and ~39.0 cm^2^ V^−1^s^−1^ (*T*_A_ = 250 °C) on high-*k* dielectrics have been reported^5^,^6^,^7^. Such results are superior to those obtained with the best RF-sputtered *In*_*2*_*O*_*3*_ TFTs, which display *μ*_e_ values of approximately 15.0 cm^2^ V^−1^ s^−1^ and an on/off current ratio (*I*_ON_/*I*_OFF_) above 10^8^ 10. At present, the highest Hall effect mobilities observed in single crystalline, polycrystalline, and amorphous *In*_*2*_*O*_*3*_ films are 160 cm^2^ V^−1^ s^−1^, 150 cm^2^ V^−1^ s^−1^, and 51 cm^2^ V^−1^ s^−1^, respectively^18^,^19^. Enhancement of the lattice ordering dimension, *i.e.*, increasing the average crystalline size, provides a fast path for carrier transport. However, two-dimensional (2D) planar defects stemming from misorientation between the crystallites may act as charge traps, resulting in the formation of a potential energy barrier that impedes the transport of free carriers. Therefore, understanding and controlling the crystalline microstructure are critical to improve charge mobility in *In*_*2*_*O*_*3*_-based TFTs. While the carrier mobility in a crystallite is relatively higher than that in an amorphous phase, there is ongoing debate as to whether *MOS* crystallites enhance charge carrier transport^6^,^7^, as grain boundaries (GBs) can serve as charge scattering sites^1^,^4^,^15^,^16^.

Efforts to partially crystallize oxide thin films have significantly improved the carrier mobility in materials systems such as *IZO* and *ZnO*^4^,^15^,^16^. Optimum crystallization of the oxide thin films was predicted to be dependent on the formation of GBs^4^,^15^,^16^. Recently, it has been reported that heterogeneous metallic seeds, *e.g.*, individual or aggregated forms of assisted *Li* atoms, could enhance the low-*T* crystallization of *ZnO* thin films, thereby yielding high-performance TFTs^15^,^16^,^20^.

In this work, ordered *In*_*2*_*O*_*3*_ films on zirconium oxide (*ZrO*_*2*_) gate dielectrics were successfully fabricated by a low-*T* (≤250 °C) solution-based processing method. It was found that the *Li*-assisted precursor films on *ZrO*_*2*_ dielectrics could form ordered structures even at processing temperatures below 200 °C. Different *In* oxidation states were observed in the *Li*-doped films, and the formation of such states was significantly affected by both temperature and the mol% of *Li* cations, [*Li*^+^]/([*In*^3+^] + [*Li*^+^]), in the precursor solutions. Upon annealing the *Li*-assisted precursor films below 200 °C, metastable indium hydroxide (*In*(*OH*)_*3*_) and/or indium oxyhydroxide (*InOOH*) phases were formed. These states were subsequently transformed into crystalline *In*_*2*_*O*_*3*_ nanostructures after further thermal dehydration and oxidation. Finally, an *In*_*2*_*O*_*3*_ film doped with 13.5 mol% *Li*^+^ and annealed at 250 °C for 1 h exhibited the highest electron mobility of 60 cm^2^ V^−1^ s^−1^ and an on/off current ratio above 10^8^ when utilized in a thin film transistor.

## Results

### Conventional High-*T* Film Fabrication of Solution-Processed *In*_*2*_ *O*_*3*_

It is known that oxide formation from *MOS* precursors based on metal acetates, nitrates, and halides is an endothermic process in which a massive external energy input is needed to form metal-O-metal lattices^5^. In many cases, a phase transition requires an elevated *T*, typically higher than 400 °C, to completely decompose the precursor and avoid undesirable organic contamination within the resulting *MOS* films. Consequently, most solution-based methods developed for the fabrication of oxide films have been incompatible with the use of plastic substrates, which have poor thermal stability and a higher thermal expansion coefficient.

In order to investigate the thermal dehydration, decomposition, and crystallization behaviors of the spun-cast films during the annealing treatment, thermogravimetric differential thermal analysis (TG-DTA) was first conducted for a powder dried from a 9 mol% *In*(*NO*_*3*_)_*3*_*·xH*_*2*_*O* solution. It should be noted that 9 mol% *In*(*NO*_*3*_)_*3*_*·xH*_*2*_*O* solutions were also used in the fabrication procedure for all thin films.

**Figure 1:**
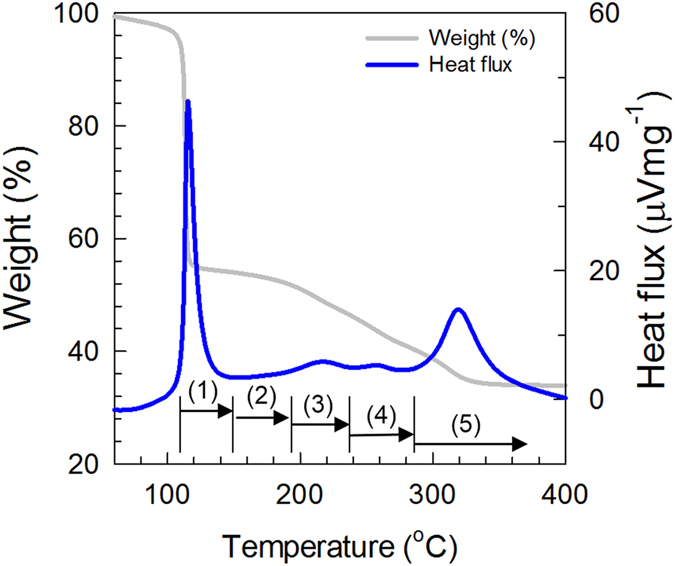
TG-DTA profiles showing weight and heat flow variations for dried *In*(*NO*_*3*_)_*3*_*·xH*_*2*_*O* powder as a function of *T*. Data were obtained at a constant heating rate of 10 °C min^−1^. The notable characteristics in each temperature zone are as follows: (**1**) transition from *In*(*NO*_*3*_)_*3*_ to *In*(*OH*)_*3*_ with a weight loss of about 45%, (**2**) *In*(*OH*)_*3*_ melting and removal of H_2_O, (**3**) removal of H_2_O from *In*(*OH*)_*3*_ with a weight loss of 10%, (**4**) conversion of *InOOH* to *In*_*2*_*O* with 7% weight loss, and (**5**) residual decomposition and crystallization with a 16% weight loss.

Figure 1 shows representative TG-DTA profiles of the dried *In*(*NO*_*3*_)_*3*_*·xH*_*2*_*O* powder, including typical weight loss behavior and heat flux variations as a function of *T*. Based on the TG-DTA results, 5% of the water residue in the dried *MOS* precursor was eliminated before decomposition. In the temperature range of 110 to 150 °C (the second heating zone in Fig. 1), a weight loss of about 45% was observed due to the hydrothermal reaction. Here, *In*(*NO*_*3*_)_*3*_ started to decompose and change into *In*(*OH*)_*3*_; this was confirmed by grazing-incidence X-ray diffraction (GIXD) analysis (as will be discussed later). In the third heating zone, endothermic melting of the *In*(*OH*)_*3*_ occurred. It has been reported that *In*(*OH*)_*3*_ can be transformed into orthorhombic *InOOH*, an intermediate product^21^,^22^,^23^. Such a reaction could be responsible for the additional 10% weight loss above 187 °C due to thermal dehydroxylation. As the temperature was raised above 275 °C, the final *In*_*2*_*O*_*3*_ product started to form via decomposition of the *InOOH*. Crystallization of the *In*_*2*_*O*_*3*_ was ultimately achieved above 400 °C^22^.

**Figure 2:**
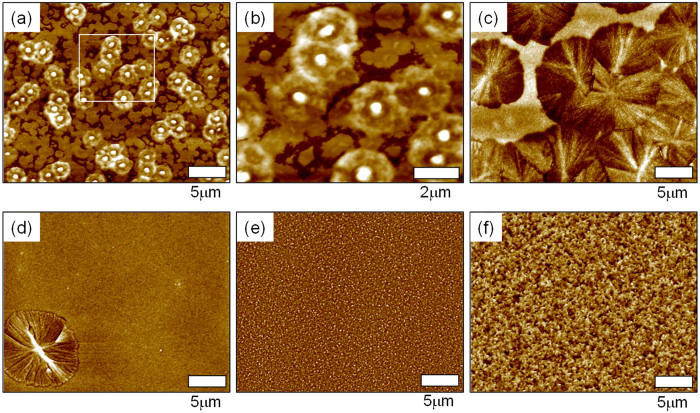
AFM topographies of Thermally annealed *In(NO*_*3*_)_*3*_ films onto *ZrO*_*2*_ surfaces. (**a,b**) 130 °C, (**c**) 170 °C, (**d**) 250 °C, (**e**) 400 °C, and (**f**) 500 °C for 1 h.

**Figure 3:**
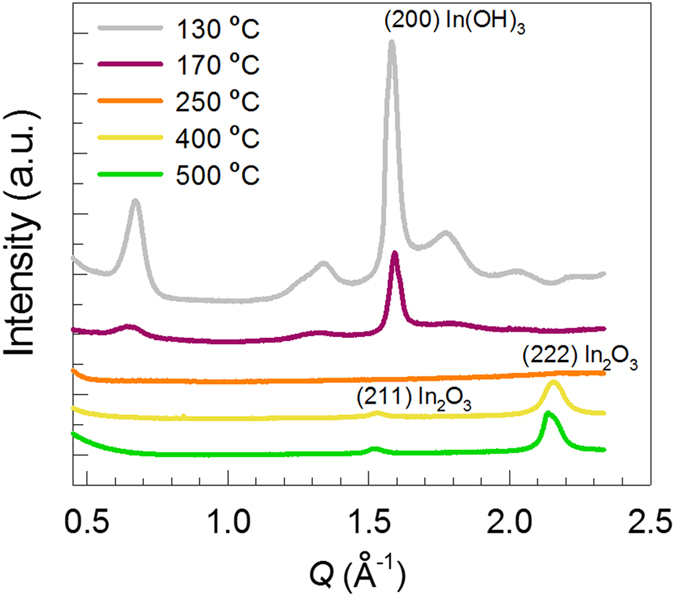


The morphologies of films annealed at a given *T* corresponding to each heating zone in Fig. 1 were systematically examined by atomic force microscopy (AFM). *MOS* precursor layers were spun-cast onto *ZrO*_*2*_*/Si* substrates from a 9 mol% *In*(*NO*_*3*_)_*3*_*·xH*_*2*_*O* solution and then thermally annealed at various temperatures for 1 h. Figure 2 shows typical AFM topographies of heat-treated precursor layers with clearly discernible phases. For the 130 °C-annealed film, flower-like agglomerates with an average diameter of 2.7 μm and height of 40−50 nm were observed. In contrast, the 170 °C-annealed film was composed of large *InOOH* grains. These spherulitic grains disappeared almost entirely in the 250 °C-annealed film. After annealing above 250 °C, micron-sized grains were completely absent, and either smooth surfaces or nano-sized aggregates were observed. The nano-sized aggregates in the 500 °C-annealed film (Fig. 2f) were confirmed to be *In*_*2*_*O*_*3*_ crystals by GIXD analysis (Fig. 3)^24^.

To determine the structure of the metal precursor films on *ZrO*_*2*_ after annealing at various *T*, GIXD data were acquired. The 1D GIXD profiles of indium oxide layers annealed at different temperatures are displayed in Fig. 3; reflections from both ordered indium oxide phases and amorphous structures are evident. The 130 °C-annealed film with flower-like crystals showed intense X-ray reflections, specifically at a scattering vector (*Q*) of 1.58 Å^−1^, which corresponded to an inter-plane distance (*d*_hkl_ = 2π/*Q*) of 3.98 Å between the (200) planes of *In*(*OH*)_*3*_ crystals. The *In*(*OH*)_*3*_ had a cubic Pn3m(224) structure with a lattice distance of 7.958 Å (ICDD PDF # 17-0549). For the 170 °C-annealed film, the intensities of the (200) reflections decreased considerably. The 1D X-ray profile of the 250 °C-annealed film did not show any clear X-ray reflections, and it was subsequently found that the film was primarily composed of *InOOH* (as determined by XPS analysis), suggesting that *In*(*OH*)_*3*_ crystallites were transformed to a less-ordered *InOOH* phase by melting and dehydration. Gurlo *et al.* reported that an aggregate form of *In*_*2*_*O*_*3*_ could be synthesized by annealing *InOOH* under ambient pressure^23^. In contrast to the other specimens, the 400 °C- and 500 °C-annealed films showed intense X-ray reflections at *Q* = 1.521 and 2.151 Å^−1^, respectively, which corresponded to the (222) and (211) crystal planes of *In*_*2*_*O*_*3*_ crystallites with an Ia-3(206) structure and a lattice distance of 10.12 Å (ICDD PDF # 06-0416). Based on the GIXD results, it was concluded that *In*_*2*_*O*_*3*_ films could be formed from solution-processed *In*(*NO*_*3*_)_*3*_*·xH*_*2*_*O* precursor layers via a solid-solid phase transition induced by high-*T* dehydration and oxidation.

### Li-assisted Solid-Solid Transformation of *In(NO*_*3*_)_*3*_ at Low Temperature

The charge carrier mobility in inorganic semiconductor films is sufficient for TFT applications, provided that a suitable fabrication method is employed^25^. Sputtered *MOS*-based TFTs generally exhibit the highest *μ*_e_ values up to 100 cm^2^ V^−1^ s^−1^. However, difficulties in terms of optimization have been encountered for most solution-processed metal precursor systems when the corresponding films were treated at low *T*^1^. As mentioned earlier, solution-processed layers from *MOS* precursors require a high-*T* treatment to remove impurities as charge trap sites and induce crystallization of the *MOS* phases for high-performance TFTs^1^.

Adamapoulos *et al.* reported that *Li*-doped *ZnO* films fabricated by an ambient solution-spray technique and annealed at 400 °C possessed a high *μ*_e_ of 85 cm^2^ V^−1^ s^−1^ in TFTs^16^. Recently, many studies have focused on the low-*T* processing of solution-based *MOS* films. *In* research by Marks and co-workers, *In*_*2*_*O*_*3*_, *ZTO*, and *IZO* TFTs were fabricated by solution-casting metal precursors and subsequently developing the structures at temperatures as low as 200 °C. Such work highlighted the benefits of exploiting self-sustaining combustion reactions to prepare *MOS* TFTs^5^. Laser irradiation has also been utilized to generate strong, localized exothermic heat so as to produce ordered *MOS* phases from precursor films at a given *T* (≤200 °C). The resulting *In*_*2*_*O*_*3*_ TFT was manipulated on transparent polymer substrates, and *μ*_e_ values of up to 6 cm^2^ V^−1^ s^−1^ were obtained. However, the device exhibited a poor *I*_ON_/*I*_OFF_ ratio of about 10^3^ 26.

**Figure 4:**
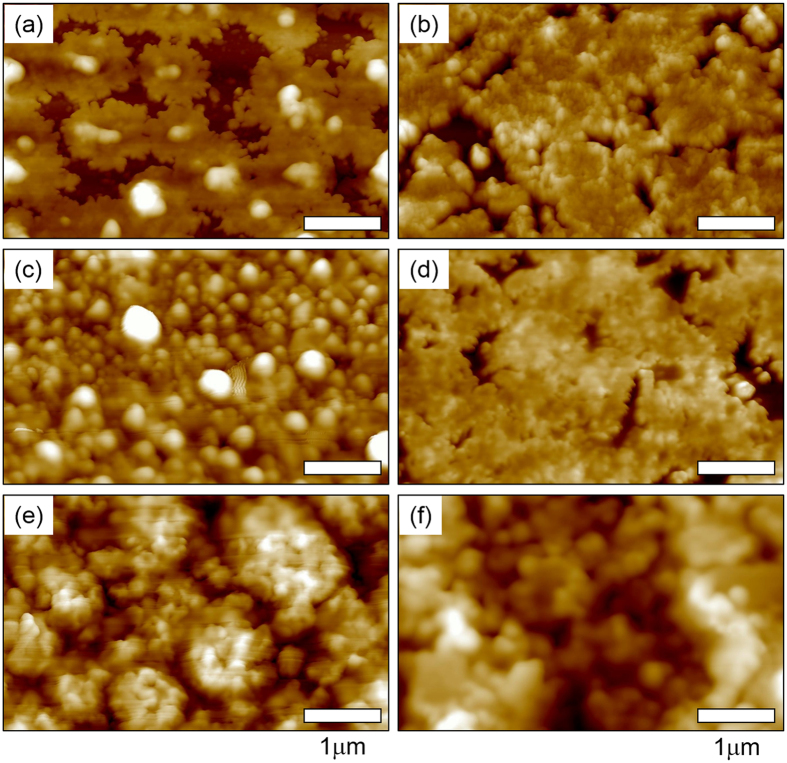
AFM topographies of 130 °C-annealed films with different *Li*^+^ loadings. (**a**) 0, (**b**) 6.7, (**c**) 8.7, (**d**) 13.5, (**e**) 21, and (**f**) 30 mol%.

To investigate the effects of *Li* incorporation on the phase transition from *In*(*NO*_*3*_)_*3*_*·xH*_*2*_*O* to *In*_*2*_*O*_*3*_, *LiNO*_*3*_-loaded *In*(*NO*_*3*_)_*3*_*·xH*_*2*_*O* solutions were prepared and spun-cast onto *ZrO*_*2*_*/Si* substrates. AFM was carried out for the *LiNO*_*3*_-assisted *In*(*NO*_*3*_)_*3*_*·xH*_*2*_*O* layers before and after thermal annealing at each annealing temperature. Figure 4 shows the AFM topographies of the 130 °C-annealed films; discernible phase morphologies are evident depending on the mol% of *Li*^+^ in the casting solutions. The existence of *LiNO*_*3*_ in these precursor layers seemed to reduce the formation of *In*(*OH*)_*3*_ flower-like aggregates in the annealed *LiNO*_*3*_-assisted films. The 6.7 mol% *Li*^+^ -loaded film appeared to be smooth with percolated grains, while the specimen prepared with a *Li*^+^ content of 8.7 mol% contained nano-sized aggregates as shown in Fig. 4b,c. Interestingly, the introduction of 13.5 mol% *Li*^+^ into the previously mixed solution produced a densely-packed layer with a height of 45−50 nm on the *ZrO*_*2*_ surface after a thermal treatment at 130 °C for 1 h (Fig. 4d). However, as the *Li*^+^ fraction was increased above 20 mol% in the solutions (or films), the *LiNO*_*3*_ and *In*(*NO*_*3*_)_*3*_ mixtures may be phase-separated during film processing, thereby causing an increase in average surface roughness (*R*_q_), as shown in Fig. 4e,f. For films prepared with 21 and 30 mol% *Li*^+^, the surface roughness was found to be 21.2 and 26.0 nm, respectively, which was much higher than those of the lower LiNO_3_-assisted films. Based on the AFM morphologies of the 130 °C-annealed films with different *Li*^+^ loadings, it is believed that, even at 130 °C, an optimized process for the incorporation of metal ions or their complexes could generate nuclei so as to facilitate the development of ordered and uniform metal oxide structures.

**Figure 5:**
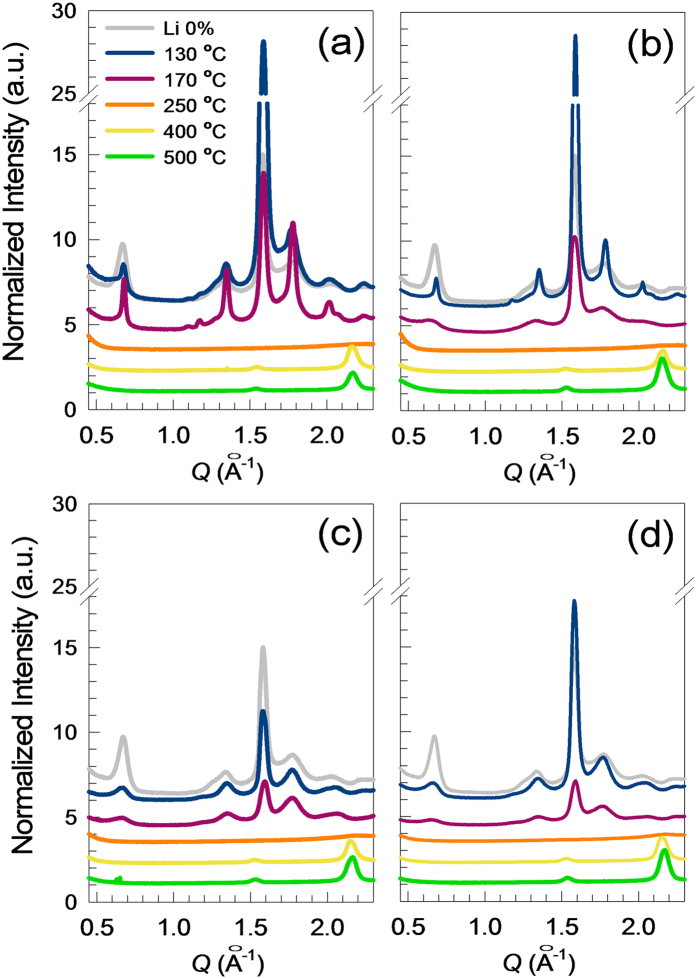
GIXD profiles of thermally annealed films with different *Li*^+^ loadings. (**a**) 6.7, (**b**) 8.7, (**c**) 13.5 and (**d**) 16.8 mol%. Profiles appearing in gray were acquired from a 130 °C-annealed film with no *Li*^+^ loading.

In order to better understand the effects of *Li* incorporation on the solid-solid phase transition in *LiNO*_*3*_/*In*(*NO*_*3*_)_*3*_ layers spun-cast onto *ZrO*_*2*_ surfaces, 2D GIXD data were obtained after annealing. Figure 5 shows the GIXD patterns of annealed films with different *Li* doping levels (the GIXD patterns of films prepared with no *Li* doping were already presented in Fig. 3). For all GIXD patterns, there was an absence of peaks associated with crystalline polymorphs induced by *Li*^+^ dopants, suggesting that *Li* may not be active in the *In*(*OH*)_*3*_, *InOOH*, and *In*_*2*_*O*_*3*_ phases.

**Figure 6:**
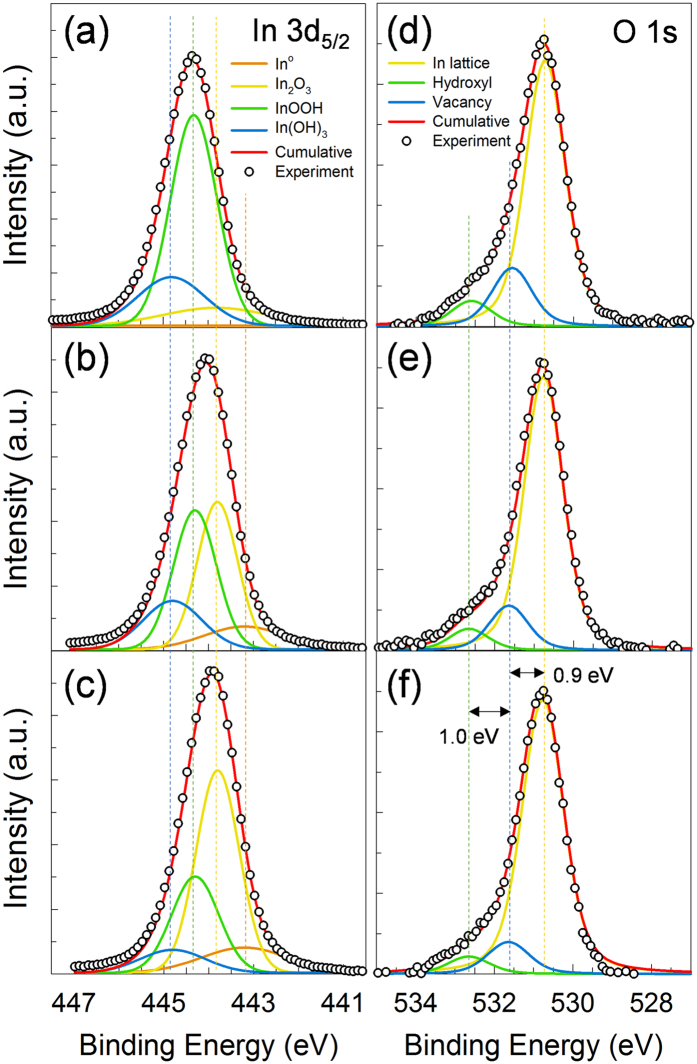
In 3d_5/2_ and *O* 1 s XPS spectra obtained for 250 °C-annealed *In*_*2*_*O*_*3*_ films with different *Li*^+^ loadings. (**a–c**) *In* 3d_5/2_ and (**d–f**) *O* 1 s, (**a,d**) 0, (**b,e**) 6.7 and (**c,f**) 13.5 mol%.

It is important to note that the X-ray reflections from *In*(*OH*)_*3*_ in the 6.7 mol% *Li*^+^-assisted film annealed at 130 °C were much more intense than those observed for samples with no *Li* doping (Fig. 5a). Based on the GIXD analysis, it is summarized that the intermediate compounds formed during In oxidation, *i.e.*, *In*(*OH*)_*3*_ and *InOOH*, quickly decayed in the annealed films, even at relatively higher *Li*^+^ loadings (see Fig. 5c), which will be addressed when discussing the X-ray photoelectron spectroscopy (XPS) findings). As shown in Fig. 5, the X-ray reflections corresponding to *In*(*OH*)_*3*_ for all films annealed at 130 °C and 170 °C, including those with *Li* dopants, tended to become weaker with an increase in the *Li*^+^ mol%. Furthermore, the patterns obtained for the 250 °C-annealed samples did not contain any reflections from *In*(*OH*)_*3*_ phases; such a trend was similar to that observed for the specimen prepared with no *Li* doping. The particular *In* oxidation states present in the XPS spectra acquired for the 250 °C-annealed films (Fig. 6) strongly support the notion that these samples are comprised of *In*(*OH*)_*3*_, *InOOH*, and *In*_*2*_*O*_*3*_. The composition ratios of these compounds changed significantly with different *Li* loadings. In contrast, the 1D GIXD profiles of the 400 °C- and 500 °C-annealed films clearly showed X-ray reflections at *Q* = 1.521 and 2.151 Å^−1^, which correspond to (211) and (222) crystal planes, respectively, in the nano-sized *In*_*2*_*O*_*3*_ crystallites (see Fig. 2e,f). The average grain sizes of *Li* doping-dependent *In*_*2*_*O*_*3*_ films annealed at 400 °C and 500 °C were calculated using X-ray diffraction profiles and Scherrer equation^27^ and summarized in Figure S1. The enhancement in average grain size of *In*_*2*_*O*_*3*_ crystal was clearly seen with increasing *Li*^+^ loadings, indicating that the incorporated *Li* can act as a catalyst for rearrangement of *In-O* bonds at the elevated temperature.

The oxidation states of *In* in the *Li*-assisted *In*_*2*_*O*_*3*_ thin films annealed at 250 °C for 1 h were systematically determined from *In* 3*d*_*5/2*_ and O 1*s* XPS spectra (see Fig. 6). Over a binding energy range of 442−446 eV, the *In* 3*d*_5/2_ spectra were found to contain contributions from *In*^*0*^, *In*_*2*_*O*_*3*_, *InOOH*, and *In*(*OH*)_*3*_ with maximum intensities at 443.2, 443.8, 444.3, and 444.8 eV, respectively^28^. Each contribution calculated from the XPS data is summarized in Table 1. As expected, the film prepared with no *Li* doping contained the highest *InOOH* fraction (0.77). As shown in Fig. 6b,c, the fraction of *InOOH* in the *Li*-assisted films decreased, while that of *In*_*2*_*O*_*3*_ at 443.8 eV increased with a rise in the *Li*^+^ mol%. Such findings indicate that *Li* incorporation can significantly enhance the oxidation of *In* in *InOOH* so as to form the desired *In*_*2*_*O*_*3*_ product for high charge carrier mobility in TFTs.

Although *InOOH* and *In*_*2*_*O*_*3*_ have similar highest occupied molecular orbital (HOMO)-lowest unoccupied molecular orbital (LUMO) band gaps of approximately 3.5 eV^29^, the stable formation of *In*_*2*_*O*_*3*_ in solution-based synthesis procedures should improve the electrical properties of *Li*-assisted films for *MOS* TFT applications. Figure 6d–f show the *O 1* *s* XPS spectra of 250 °C-annealed films with different *Li* loadings. The oxygen signals were deconvoluted into three sub-profiles with different maximum intensities at 530.8, 531.7, and 532.7 eV, respectively. The *O 1s* peaks centered at 530.8 and 531.7 eV were assigned to oxygen fully coordinated (denoted as *O*) and insufficiently coordinated (referred to as an oxygen vacancy, *V*_o_) by *In* ions, respectively^30^. Furthermore, the peak at 532.7 eV was attributed to oxygen impurities, such as hydroxyl (OH) groups. The *V*_o_-related fractions in the *O 1* *s* spectra decreased with increasing *Li* incorporation (Table 1). As the *Li* loading in the 250 °C-annealed films was increased from 0 to 13.5 mol%, the ratio of the *V*_o_-related signals to the entire XPS profile decreased from 0.167 to 0.095. The obtained results suggest that the assisted *Li* efficiently improved the coordination of *In-O* bonding so as to form energetically stable configurations. Such a scenario presumably occurred because the smaller ionic radius of *Li* allowed for improved oxygen diffusivity in the indium oxide network. Notably, the fraction of *OH* in the *Li*-assisted films was substantially lower than that in the undoped film, as shown in Fig. 6d–f and Table 1. In addition, undesirable impurities such as nitrogen and carbon were not detected in the XPS spectra (see Figure S2 in Supporting Information) of any 250 °C-treated film, regardless of the *Li* loading.

**Figure 7:**
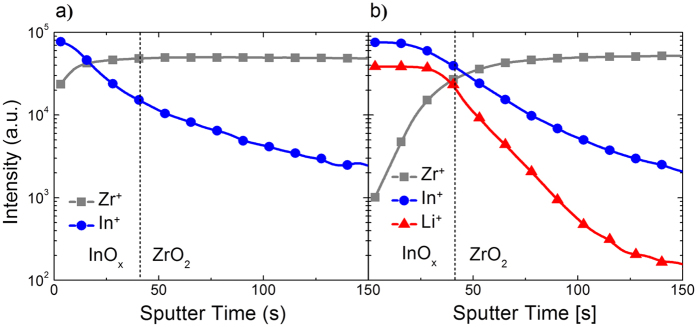
Depth profile of *Li, In*, and *Zr* cations for the *In*_*2*_*O*_*3*_/*ZrO*_*2*_ stack annealed at 250 °C with different *Li*^+^ loadings. (**a**) 0 and (**b**) 13.5 mol%.

The depth profile of incorporated *Li* in the *In*_*2*_*O*_*3*_/*ZrO*_*2*_ stack was further analyzed using the time-of-flight secondary ion mass spectroscopy (TOF-SIMS). In the *In*_*2*_*O*_*3*_/*ZrO*_*2*_ stack with no *Li* loading, the *Zr* cations in the *ZrO*_*2*_ dielectric film diffused substantially into *In*_*2*_*O*_*3*_ film during the thermal annealing at 250 °C (see Fig. 7a). The penetration of *Zr* cation was suppressed for the *In*_*2*_*O*_*3*_/*ZrO*_*2*_ stack with 13.5 mol% *Li* loading (see Fig. 7b). It suggests that the *Li*-assisted *In*_*2*_*O*_*3*_ film has the more uniform morphology and higher packing density compared to the undoped *In*_*2*_*O*_*3*_ film, which will be discussed later. It is also noted that the *Li* cation existed uniformly in the *In*_*2*_*O*_*3*_ film along depth direction. These beneficial effects of *Li* incorporation into the *In*_*2*_*O*_*3*_ films in terms of the impurity concentration should lead to superior electrical properties for the resulting TFTs.

### Electrical Properties of *Li*-assisted *In*_*2*_ *O*_*3*_ Films

**Figure 8:**
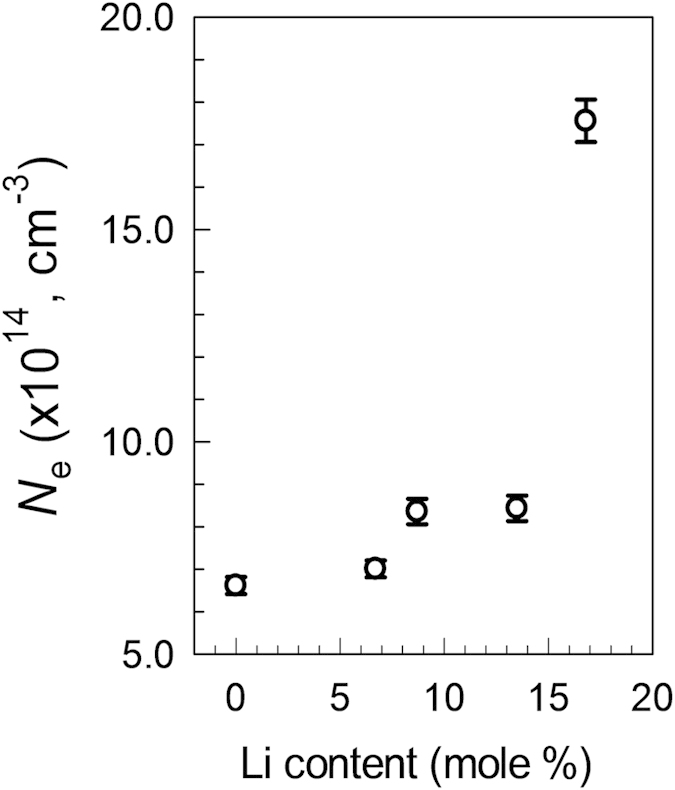
Variations in the free electron density of *In*_*2*_*O*_*3*_ thin films annealed at 250 °C as a function of Li incorporation.

The free electron concentration (*N*_*e*_) of 250 °C-annealed *In*_*2*_*O*_*3*_ films on 23-nm-thick *ZrO*_*2*_*/Si* substrates as a function of *Li* loading was determined from an analysis of *C*^−2^ vs. *V* data; the results are shown Fig. 8 31. While a slightly higher free electron concentration was produced with an increase in *Li* loading up to 13.5 mol%, a more significant rise in *N*_*e*_ was observed as the *Li* fraction was increased to 16.8 mol%. In particular, *N*_*e*_ values of 6.6−8.4 × 10^14^ and 1.8 × 10^15^ cm^−3^ were obtained for *Li*-assisted *In*_*2*_*O*_*3*_ films with 0–13.5 and 16.8 mol% *Li*, respectively. The *N*_*e*_ value is affected by the net energy difference between shallow donor states and localized trap densities. Provided that the overall donor state density is invariant, the *Li*-driven enhancement in *N*_*e*_ is mainly related to morphological changes that occur in the films as a result of *Li* incorporation.

**Figure 9:**
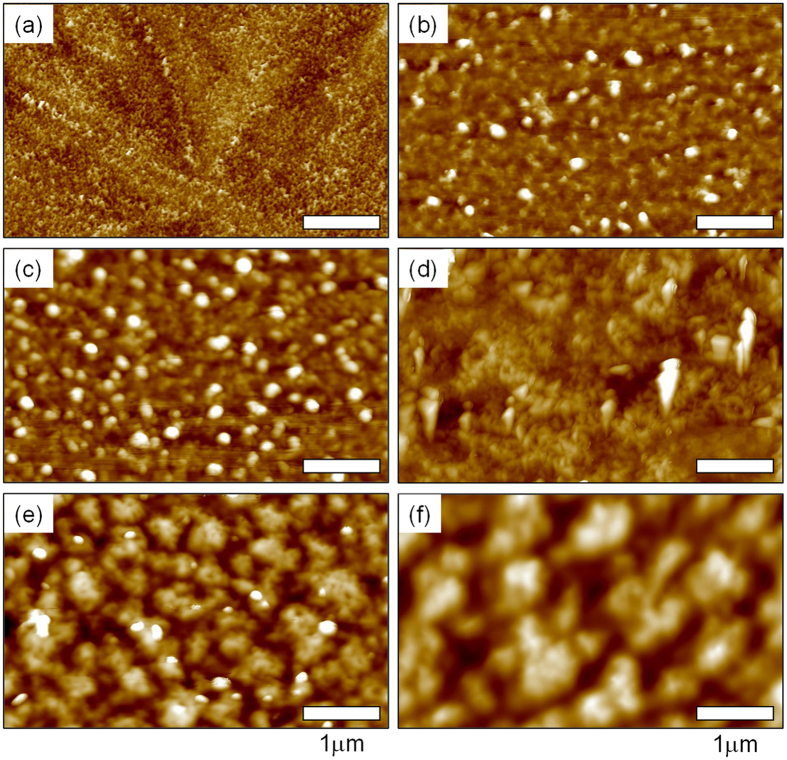
AFM topographies of 250 °C-annealed films with different *Li*^+^ loadings. (**a**) 0, (**b**) 6.7, (**c**) 8.7, (**d**) 13.5, (**e**) 16.8 and (**f**) 21 mol%.

The AFM topographies of the 250 °C-annealed *In*_*2*_*O*_*3*_ films used for the *N*_*e*_ analysis are displayed in Fig. 9. With the exception of morphological traces left by the previously-grown spherulite, no clear texture was observed in the film prepared with no *Li* doping (see Fig. 9a). As a higher mol% of *Li* was assisted, isolated nanoparticles, closely-packed grains, phase-separated domains, and bi-continuous phases were formed after annealing at 250 °C (see Fig. 9b–f). The AFM topologies of films with 6.7 and 8.6 mol% *Li*^+^ showed well-dispersed nanoparticles with sizes of 20 to 80 nm (Fig. 9b,c). These isolated nanoparticles disappeared almost entirely in the 13.5 mol% *Li*^+^-assisted film, which contained closely-packed nano-grains (Fig. 9d). With an increase in the *Li* loading above 13.5 mol% *Li*^+^, phase-separated domains were observed in the films, and their sizes increased with a rise in the *Li* mol%. The obtained results suggest that *Li* incorporation minimizes the concentration of localized trap states, including tail states and deep-level traps.

### *Li*-assisted *In*_*2*_ *O*_*3*_ TFTs on Solution-Derived *ZrO*_*2*_ Dielectrics

**Figure 10:**
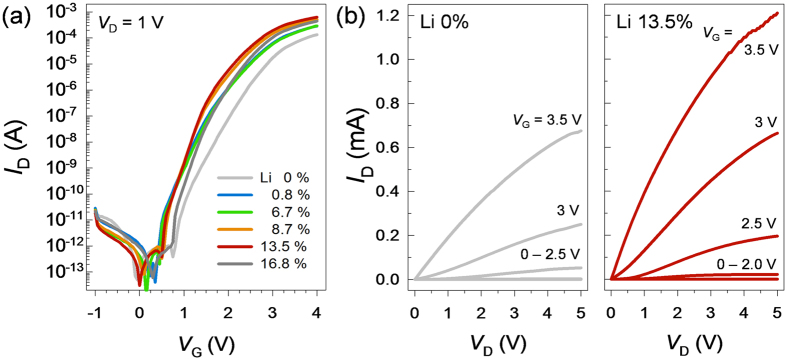
Electrical Properties of *Li*-assisted *In*_*2*_*O*_*3*_ TFTs with different Li loadings. (**a**) Transfer characteristics and (**b**) output characteristics.

Typical *I*_D_-*V*_G_ transfer characteristics of *Li*-assisted *In*_*2*_*O*_*3*_ TFTs annealed at 250 °C are shown in Fig. 10; the corresponding electrical properties are summarized in Table 2. The subthreshold swing (*SS*) was extracted from a linear region of the log(*I*_D_)-*V*_G_ plot. The densities of fast bulk traps (*N*_*SS*_) and semiconductor-insulator interface traps (*D*_*it*_) were calculated using the following expression^32^:


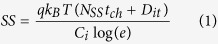


where *q* is the electron charge, *k*_*B*_ is Boltzmann’s constant, *T* is the absolute temperature, and *t*_*ch*_ is the channel layer thickness. Maximum values of *N*_*SS*_ and *D*_*it*_ were derived by setting one of the parameters to zero.

For the *In*_*2*_*O*_*3*_ TFT prepared with no *Li* doping, *μ*_*e*_, *V*_*th*_, and *SS* values of 19.4 ± 0.1 cm^2^ V^−1^ s^−1^, 2.48 V, and 0.33 V decade^−1^, respectively, were obtained along with an *I*_ON_/*I*_OFF_ ratio of 1.34 × 10^8^. The high *I*_ON_/*I*_OFF_ ratio achieved on the solution-processed *ZrO*_*2*_ gate dielectric could be the result of moderate *N*_*e*_ values in the patterned *In*_*2*_*O*_*3*_ film. Interestingly, the *μ*_*e*_ value of the *Li*-assisted *In*_*2*_*O*_*3*_ TFTs was enhanced substantially by increasing the *Li*^+^ mol% in the channel layer. In particular, the average *μ*_*e*_ values of *Li*-assisted *In*_*2*_*O*_*3*_ TFTs with 0.8, 6.7, 8.7, 13.5, and 16.8 mol% *Li*^+^ were 33.6, 41.1, 51.1, 59.8, and 57.3 cm^2^ V^−1^ s^−1^, respectively. Furthermore, *I*_ON_/*I*_OFF_ values greater than 10^8^ were observed. The *Li*-assisted *In*_*2*_*O*_*3*_ TFT with 13.5 mol% *Li*^+^ showed the highest *μ*_*e*_ value of 60 cm^2^ V^−1^ s^−1^ and an *I*_ON_/*I*_OFF_ ratio of ~6.0 × 10^8^. Such values are comparable to those reported for state-of-the-art metal oxide TFTs fabricated by vacuum-based processes^33^. The enhanced carrier transport properties of the *Li*-assisted *In*_*2*_*O*_*3*_ TFTs when compared to the undoped *In*_*2*_*O*_*3*_ TFT are reflected in the excellent output characteristics of the devices, as shown in Fig. 10b.

Based on the TG-DTA, AFM, GIXD, and XPS findings, the three-fold increase in the value of *μ*_*e*_ for the 13.5 mol% *Li*-assisted TFT, when compared to that in the undoped device, is attributed to both an efficient phase transition from metastable *InOOH* to stable *In*_*2*_*O*_*3*_ and a densely packed film morphology after annealing at 250 °C. It is interesting to compare the *N*_*SS,max*_ and *D*_*it,max*_ values of the undoped and *Li*-assisted devices because they are likely to trap free electrons and thus, impede the electric field-driven drift velocity of the free carriers. For the *Li*-assisted *In*_*2*_*O*_*3*_ TFTs, *N*_*SS,max*_ or *D*_*it,max*_ were found to decrease at higher *Li* loadings up to 13.5 mol%. Residual *V*_o_ and/or impurities such as OH generally act as trapping centers for charge carriers, and the decrease in *N*_*SS,max*_ or *D*_*it,max*_ values at higher *Li* fractions may partially be attributed to a reduction in *V*_o_ and unwanted impurities after *Li* incorporation. However, the ~3-fold increase in *μ*_e_ for the 13.5 mol% *Li*-assisted device when compared to that of the undoped device cannot be completely explained by a ~2-fold decrease in *N*_*SS,max*_ or D_*it,max*_. It can be inferred that the effective mass of electrons in *In*_*2*_*O*_*3*_ is smaller than that in *InOOH*, although the electronic band structure of metastable *InOOH* has not yet been explicitly calculated.

**Figure 11:**
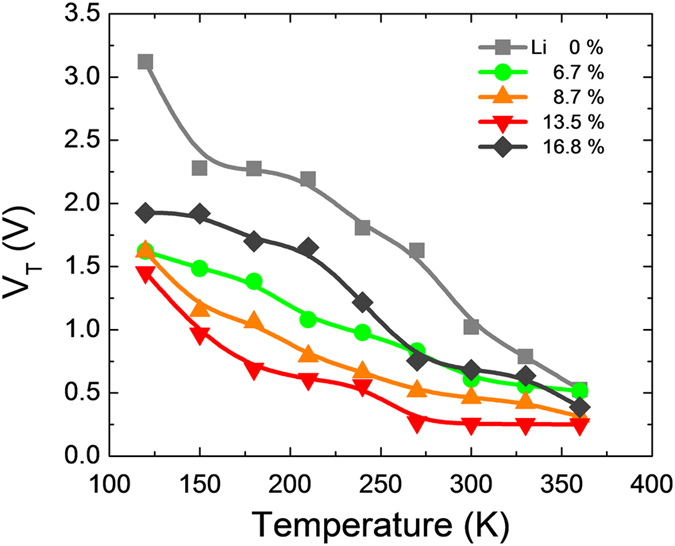
Temperature instability in terms of *V*_*th*_ values for the *In*_*2*_*O*_*3*_ TFTs with different *Li* loadings.

Finally, the thermal instability of *Li*-assisted *In*_*2*_*O*_*3*_ TFTs was examined in the temperature range from 120 to 360 K. The on-state drain current for *In*_*2*_*O*_*3*_ TFTs with no *Li* loading exhibited the thermally activated behavior with increasing measurement temperature, which resulted in the huge negative *V*_*th*_ displacement (Δ*V*_*th*_ = −2.6 V) as shown in Figure S3 and Fig. 11. This behavior has been frequently reported for the metal oxide TFTs, which can be attributed to the existence of the bulk traps and semiconductor-insulator interface traps^34^,^35^. In contrast, the *In*_*2*_*O*_*3*_ TFTs with 13.5 mol% *Li*^+^ exhibited the improved thermal stability (see Figure S3 and Fig. 11), which is consistent with the fact that the incorporated *Li*^+^ reduced the structural defect and impurity, leading to the reduction in *N*_*SS,max*_ and *D*_*it,max*_ values.

In summary, *Li*-assisted *In*_*2*_*O*_*3*_ channel TFTs fabricated on *ZrO*_*2*_ dielectrics by a low-temperature (250 °C) solution-based process exhibited superior mobilities and *I*_ON_/*I*_OFF_ ratios. It was determined that *Li* incorporation played various important roles in the *In*_*2*_*O*_*3*_*/ZrO*_*2*_ structures, including: 1) accelerating the decomposition of metastable *In*(*OH*)_*3*_ and *InOOH* phases into *In*_*2*_*O*_*3*_, 2) reducing the bulk and interface trap density in the *ZrO*_*2*_ dielectric by eliminating hydroxyl groups and oxygen vacancies, and 3) enhancing the nucleation and crystallization of *In*(*OH*)_*3*_ and *In*_*2*_*O*_*3*_ crystallites by filling interstitial sites. The use of a precursor with a high *Li* mol%, in excess of the optimum 13.5 mol% determined in this work, may cause phase separation and severe surface roughening of *LiNO*_*3*_ and *In*(*NO*_*3*_)_*3*_*-*related complexes (as inferred from the AFM findings). This in turn could increase the trap density and thus, reduce the carrier mobility.

The solution-based, low-temperature preparation procedure detailed in this report involves the simple physical blending of soluble metal and dopant precursors. As such, the devised synthesis method can expand the possibilities for the development of high-quality multi-component oxide semiconductors that can be implemented on large-area substrates.

## Methods

### Materials

Zirconium oxynitrate hydrate (*ZrO*(*NO*_*3*_)_*2*_*·xH*_*2*_*O*), *In*(*NO*_*3*_)_*3*_*·xH*_*2*_*O*, and *LiNO*_*3*_ (all purchased from Aldrich) were employed as dielectric, *MOS*, and dopant precursors, respectively, while 2-methoxyethanol (2MeEtOH, Aldrich) was utilized as a solvent. A 100-nm-thick SiO_2_ layer on a highly doped *p*-type Si substrate was used to fabricate a coplanar bottom gate. A 0.1 M *ZrO*_*2*_ precursor solution was first prepared by dissolving *ZrO*(*NO*_*3*_)(*NO*_*3*_)_*2*_*·xH*_*2*_*O* in *2MeEtOH* with stirring at room temperature for 12 h. Next, 0.3 M *MOS* precursor solutions were made by dissolving *In*(*NO*_*3*_)_*3*_*·xH*_*2*_*O* in *2MeEtOH* with stirring at 40 °C for 4 h. Different amounts of *LiNO*_*3*_ as an additive were then introduced to the *In*_*2*_*O*_*3*_ precursor solutions with stirring at 40 °C for 2 h; the *Li* fraction was varied from 0 to 30 mol% (stoichiometry in solution). All solutions were filtered through a 0.2 μm membrane-syringe filter prior to solution casting.

### Sample Preparation

In order to fabricate *Li*-assisted *In*_*2*_*O*_*3*_ TFTs with a coplanar bottom-gate and bottom-contact electrode structure (Figure S5 in Supporting Information), AZ 9200 photoresist (PR) layers were cast onto the 100-nm-thick SiO_2_/Si substrates and patterned with lines. The PR patterned SiO_2_/Si substrates were then inserted into a buffer oxide etchant to selectively remove the exposed SiO_2_ surfaces. A dielectric layer was spun-cast onto the patterned SiO_2_/Si substrates from a 0.1 M *ZrO*(*NO*_*3*_)_*2*_*·xH*_*2*_*O* solution and subsequently annealed via a two-step procedure at 100 °C for 10 min and then 250 °C for 1 h. The dielectric coating process was repeated so as to produce a *ZrO*_*2*_ film with a thickness of approximately 23 nm.

Indium tin oxide (*ITO*) source/drain (*S*/*D*) electrodes with a thickness of 150 nm were deposited on the *ZrO*_*2*_ layer via sputtering of an *ITO* target with 90% *In*_*2*_*O*_*3*_. The ITO was then patterned by PR coating/developing and *ITO* etching in a dilute HCl solution. The dimensions of the patterned *ITO* pads in the TFTs were controlled so as to ensure a channel length (*L*) and width (*W*) of 14 μm and 150 μm, respectively. Different *Li*-assisted *In*_*2*_*O*_*3*_ precursor layers were subsequently spin-cast onto the patterned *ITO*/*ZrO*_*2*_/*Si* substrates and annealed at 100 °C for 10 min. Finally, the samples were loaded into a box furnace and thermally annealed at different *T* from 130 °C to 600 °C for 1 h; the heating rate was 2.5 °C min^−1^. It should be noted that all samples for X-ray and morphological characterization were fabricated on unpatterned *ZrO*_*2*_*/Si* substrates.

### Characterization

The dehydration, decomposition, and crystallization kinetics of dried *LiNO*_*3*_, *In*(*NO*_*3*_)_*3*_*·xH*_*2*_*O*, and mixed powders were investigated from 25 °C to 600 °C using TG-DTA (TG 209 F3 Tarsus^®^, NETZSCH) with a heating rate of 10 °C min^−1^ from 25 °C to 600 °C under an air ambient condition. Film thicknesses were calculated from the corresponding synchrotron-based X-ray reflectivity (XRR, beamline X9, Brookhaven National Laboratory, USA) profiles. XPS (K-Alpha Thermal Scientific) was performed with *K*_α_ radiation so as to investigate the elemental chemistry and bonding in the *Li*-assisted *In*_*2*_*O*_*3*_ thin films. AFM (Multimode 8, Bruker) was carried out to examine the nano-structural morphologies of the fabricated samples. The crystalline structure of the films was evaluated by synchrotron-based GIXD beamlines 3 C and 9 A, Pohang Acceleration Laboratory, Korea^36^,^37^.

The electrical characteristics of the *Li*-assisted *In*_*2*_*O*_*3*_ TFTs were measured with a semiconductor analyzer (Agilent 4155 C). The electron mobility (*μ*_e_) and threshold voltage (*V*_th_) values were calculated in the saturation regime (drain voltage, *V*_D_ = 1 V) using the following equation, *I*_D_ = *μ*_e_*C*_i_*W*(2*L*)^−1^(*V*_G_–*V*_th_)^2^, where *C*_i_ is the capacitance of the gate dielectrics and *V*_G_ is the gate voltage. The *C*_i_ values of the dielectrics, which were sandwiched between the *ITO* and highly doped *p*-type (100) Si substrate, were measured with an Agilent E4980A instrument.

## Additional Information

**How to cite this article**: Nguyen, M.-C. *et al.* Li-Assisted Low-Temperature Phase Transitions in Solution-Processed Indium Oxide Films for High-Performance Thin Film Transistor. *Sci. Rep.* **6**, 25079; doi: 10.1038/srep25079 (2016).

## Supplementary Material

Supplementary Information

## Figures and Tables

**Table 1:** Variations in the oxidation state of *In 3d*_*5/2*_ and *O 1 s* XPS peaks for 250 °C-annealed *In*_*2*_*O*_*3*_ films with different *Li* loadings.

*Li*^* *+* *^[mol%]	*In*^0^	*In*(*OH*)_*3*_	*InOOH*	*In*_*2*_*O*_*3*_	[*O*]	[*V*_o_]	[*OH*]
0	0.05	0.12	0.77	0.06	0.76	0.17	0.07
8.7	0.08	0.17	0.38	0.37	0.81	0.13	0.06
13.5	0.10	0.12	0.36	0.42	0.85	0.10	0.05

**Table 2:** Electrical properties of *Li*-assisted *In*_*2*_*O*_*3*_-based TFTs with various *Li* loadings on 23 nm-thick *ZrO*_*2*_ dielectric layers.

*Li*^+^ [mol%]	*V*_th_ [V]	*SS*[V dec^−1^]	*I*_ON_/*I*_OFF_ [10^8^]	*μ*_e_ [cm^2^ V^−1^ s^−1^]	*D*_it,max_ [10^12^ eV^−1^ cm^−2^]	*N*_SS, max_ [10^18^ eV^−1^ cm^−3^]
0	2.48	0.33	1.34	19.4 ± 0.1	2.4	4.8
0.8	2.33	0.29	2.84	33.6 ± 0.1	2.1	4.3
6.7	2.26	0.25	2.92	41.1 ± 0.1	1.8	3.7
8.7	2.11	0.19	5.09	51.1 ± 0.2	1.4	2.8
13.5	2.02	0.18	6.30	59.8 ± 0.2	1.3	2.6
16.8	2.23	0.2	4.45	57.3 ± 0.2	1.5	2.9
