# Supplementary material for: Li-Assisted Low-Temperature Phase Transitions in Solution-Processed Indium Oxide Films for High-Performance Thin Film Transistor
Source: Sci Rep. 2016 Apr 28;6:25079. doi: 10.1038/srep25079 (PMC4848541; doi:10.1038/srep25079)
Supplement: Supplementary Information [file srep25079-s1.doc]

Supporting Information

Li-Assisted Low-Temperature Phase Transitions in Solution-Processed Indium Oxide Films for High-Performance Thin Film Transistor

Manh-Cuong Nguyen^1^, Mi Jang^2^, Dong-Hwi Lee^1^, Hyun-Jiun Bang^1^, Minjung Lee^2^, Jae Kyeong Jeong^3^*, Hoichang Yang^2^* & Rino Choi^1^*

^1^Department of Materials Science and Engineering, Inha University, Incheon 402-751, Republic of Korea, ^2^Department of Applied Organic Materials Engineering, Inha University, Incheon 402-751, Republic of Korea, ^3^Department of Electronic Engineering, Hanyang University, Seoul 133-791, Republic of Korea

**
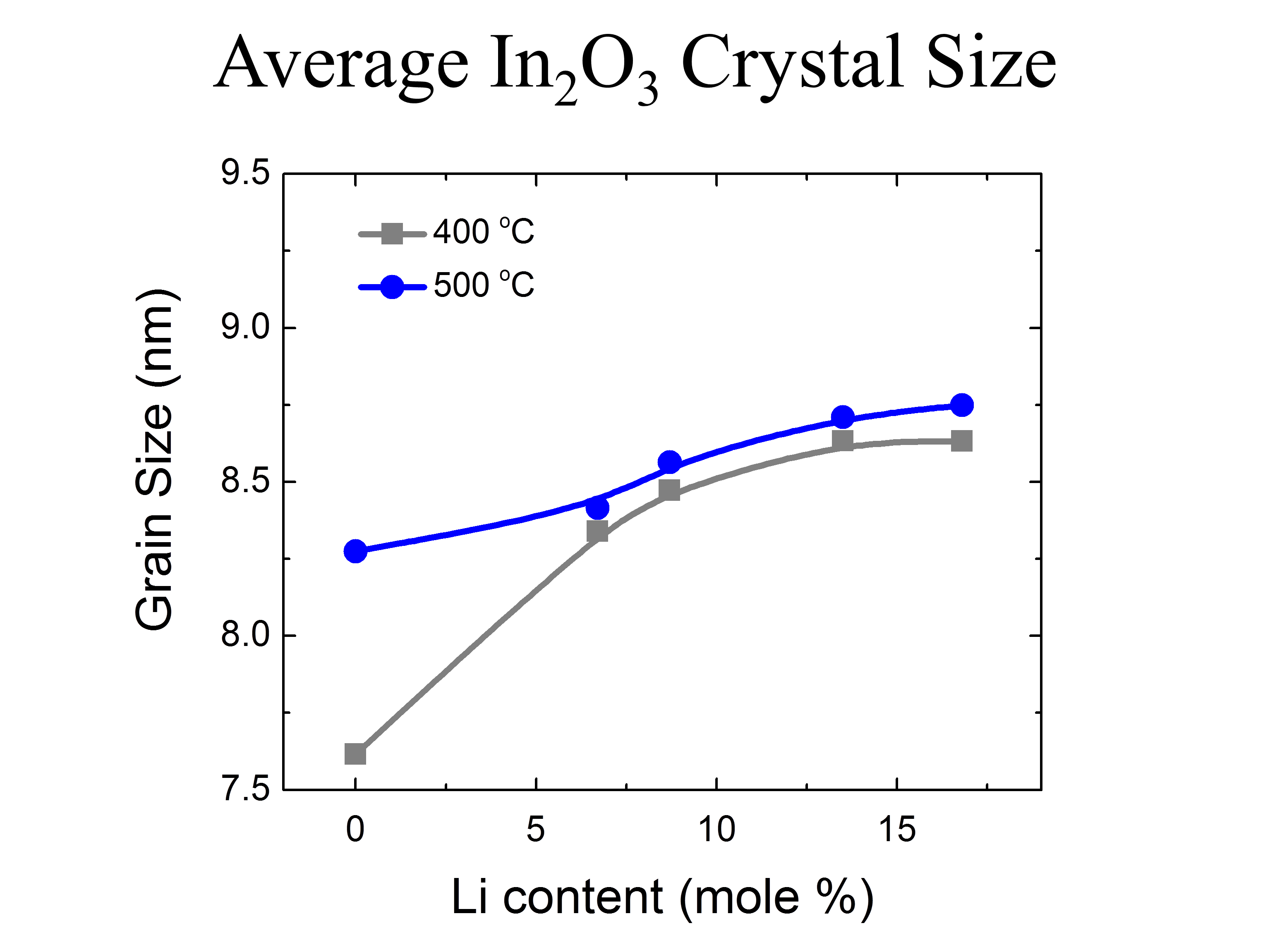
**

**Figure S1.** Average crystal size of Li-assisted In_2_O_3_ annealed at 400 ^o^C and 500 ^o^C calculated by Scherrer equation.

**Figure S2.** (a) *C* 1s and (b) *N* 1s XPS spectra obtained for 250 °C-annealed *In_2_O_3_* films with different *Li*^+^ loading of 0, 6.7, 13.5 mol%.


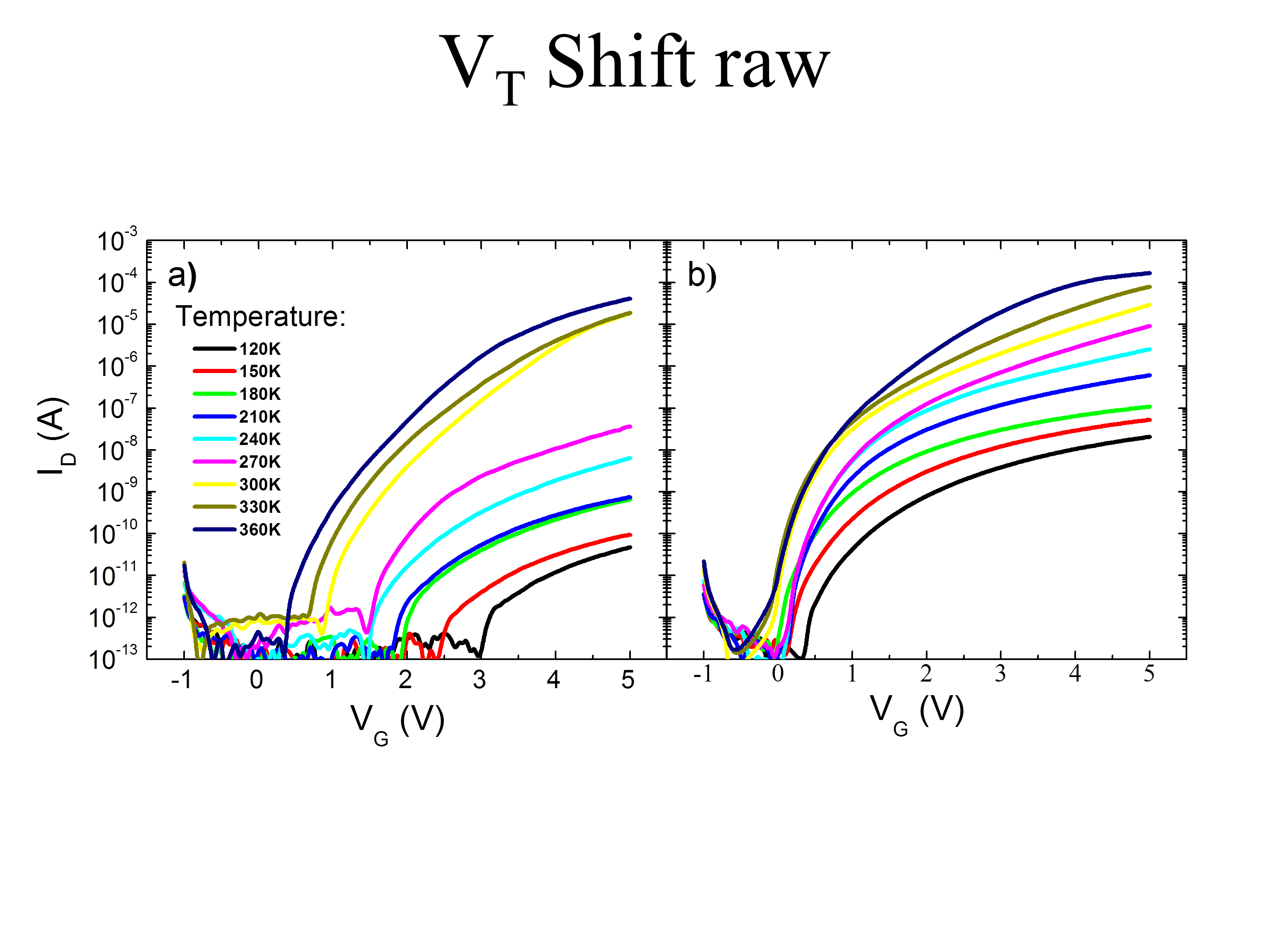


**Figure S3.** Thermal stability of *Li*-assisted *In_2_O_3_* TFTs with and without *Li*^+^ loading: (a) 0, and (b) 13.5 mol%.

**Figure S4.** Fabrication schemes for the coplanar bottom-gate and bottom-contact electrodes in the *In_2_O_3_*-based TFTs: (1) PR patterning, (2) *SiO_2_* patterning, (3) *ZrO_2_* spin-casting, (4) *ITO* sputtering and patterning, and (5) metal precursor spin-casting and heating. All active channels were patterned by etching the *Li*-assisted *In_2_O_3_* layers in a 1% HCl solution for 10 s.
